# Digital Health Interventions and Patient Safety in Abdominal Surgery: A Systematic Review and Meta-Analysis

**DOI:** 10.1001/jamanetworkopen.2024.8555

**Published:** 2024-04-26

**Authors:** Artem Grygorian, Diego Montano, Mahdieh Shojaa, Maximilian Ferencak, Norbert Schmitz

**Affiliations:** 1 Faculty of Medicine, Department of Population-Based Medicine, Tuebingen University, Tuebingen, Germany; 2 Faculty of Medicine, Department of Psychiatry, McGill University, Montreal, Québec, Canada

## Abstract

**Importance:**

Over the past 2 decades, several digital technology applications have been used to improve clinical outcomes after abdominal surgery. The extent to which these telemedicine interventions are associated with improved patient safety outcomes has not been assessed in systematic and meta-analytic reviews.

**Objective:**

To estimate the implications of telemedicine interventions for complication and readmission rates in a population of patients with abdominal surgery.

**Data Sources:**

PubMed, Cochrane Library, and Web of Science databases were queried to identify relevant randomized clinical trials (RCTs) and nonrandomized studies published from inception through February 2023 that compared perioperative telemedicine interventions with conventional care and reported at least 1 patient safety outcome.

**Study Selection:**

Two reviewers independently screened the titles and abstracts to exclude irrelevant studies as well as assessed the full-text articles for eligibility. After exclusions, 11 RCTs and 8 cohort studies were included in the systematic review and meta-analysis and 7 were included in the narrative review.

**Data Extraction and Synthesis:**

Data were extracted according to the Preferred Reporting Items for Systematic Reviews and Meta-Analyses (PRISMA) reporting guideline and assessed for risk of bias by 2 reviewers. Meta-analytic estimates were obtained in random-effects models.

**Main Outcomes and Measures:**

Number of complications, emergency department (ED) visits, and readmissions.

**Results:**

A total of 19 studies (11 RCTs and 8 cohort studies) with 10 536 patients were included. The pooled risk ratio (RR) estimates associated with ED visits (RR, 0.78; 95% CI, 0.65-0.94) and readmissions (RR, 0.67; 95% CI, 0.58-0.78) favored the telemedicine group. There was no significant difference in the risk of complications between patients in the telemedicine and conventional care groups (RR, 1.05; 95% CI, 0.77-1.43).

**Conclusions and Relevance:**

Findings of this systematic review and meta-analysis suggest that perioperative telehealth interventions are associated with reduced risk of readmissions and ED visits after abdominal surgery. However, the mechanisms of action for specific types of abdominal surgery are still largely unknown and warrant further research.

## Key Points

**Question:**

Can perioperative telemedicine reduce the incidence of adverse events in abdominal surgery?

**Findings:**

In this systematic review and meta-analysis of 19 randomized trials and nonrandomized studies with 10 536 patients, significant reduction in readmission rates and emergency department visits was found with telemedicine interventions compared with conventional care after abdominal surgery. There was no difference in complication rates between patients in telehealth and conventional care groups.

**Meaning:**

Findings of this study suggest that perioperative telemedicine may play a role in reduced incidence of readmissions and emergency department visits after abdominal surgery; however, further research is needed to elucidate specific mechanisms of action.

## Teaser

This systematic review and meta-analysis evaluates the complication, emergency department, and hospital readmission rates associated with telemedicine vs conventional care in patients who underwent abdominal surgery.

## Introduction

The past decade has been marked by the widespread use of digital health technologies. Limited access to health care resources, mobility restrictions of individuals, and disruptions in the provision of health services during pandemics have encouraged the adoption of telehealth technologies in previously limited areas, such as surgery.^1^ The diversity of digital health interventions and the variety of applications in surgery make an overall assessment of benefits and effectiveness difficult. Most available studies have focused on assessing patient satisfaction and clinician experience as well as time and cost savings related to telemedicine interventions.^2,3^

Abdominal and pelvic surgery (including colorectal and gynecological) is one of the most common types of surgery, with its own specific complications and recovery conditions. Due to its potential to reduce the delay between complication onset and medical intervention, telemedicine may facilitate the early detection of postoperative complications by providing options for patient-clinician communication and remote patient monitoring.^2,3^ In general, telemedicine may be used as an instrument of patient safety that can identify and prevent health hazards from the provision of health services.^4^ Although patient safety can be assessed with different indicators, the most frequent indicators may include complications, readmissions, emergency department (ED) visits, nosocomial infections, and adverse events.

Despite the increasing number of telemedicine interventions in abdominal surgery, to our knowledge, the implications of digital health interventions for patient safety in abdominal surgery have not been quantitatively assessed. Therefore, in this systematic review and meta-analysis, we aimed to estimate the implications of telemedicine interventions for complication and readmission rates in a population of patients with abdominal surgery.

## Methods

The review protocol was registered in PROSPERO (identifier: CRD42022338147). We followed the Preferred Reporting Items for Systematic Reviews and Meta-analyses (PRISMA) reporting guideline.

### Eligibility Criteria

Articles were included in this meta-analysis if they met the following inclusion criteria: (1) randomized clinical trials (RCTs), nonrandomized controlled trials, and observational (cohort and case-control) studies; (2) adult patients with abdominal and pelvic surgical procedures (including colorectal, gynecological, and urological), without sex or any age restrictions; (3) digital health interventions provided in the preoperative and postoperative periods, including remote consultations via video or telephone call, digital follow-up and recovery programs delivered via a mobile application, and remote monitoring of health status; and (4) at least 1 of the reported primary outcomes related to patient safety, including complications and adverse events, readmissions, and ED visits. The exclusion criteria were (1) studies without a comparable group; (2) other types of digital health interventions without patient-clinician communication; (3) studies reporting less than 2 outcomes measurement; and (4) reviews, commentaries, letters, editorials, case reports, and non-peer-reviewed sources.

Following the World Health Organization^5^ classification, we categorized the studies into 4 types of interventions: health care consultations between patient and clinician, remote monitoring of patient health or diagnostic data by clinician, transmission of medical data to clinician, and case management consultations between clinicians.

### Search Strategy and Selection Process

A systematic literature search was conducted of MEDLINE (via PubMed and Ovid), Cochrane Library, and Web of Science databases for all articles published up to February 2, 2023. The search strategy was based on types of telemedicine interventions, outcomes, and population. The terms *telemedicine*, *patient safety*, and *surgery* were the basic concepts used to specify the query strings for the databases. Keywords and MeSH (Medical Subject Headings) terms were used to refine the database queries. A search strategy developed for PubMed is provided in the eAppendix in Supplement 1. This protocol was applied to all database searches, with some modifications to search terms and operators.

Two of us (A.G., M.F.) independently screened the titles and abstracts obtained from the searches to exclude irrelevant studies. After the interrater reliability was deemed appropriate (κ = 0.998), 2 of us (A.G., M.F.) separately and independently assessed the full-text articles for eligibility against the predetermined inclusion criteria. Any disagreements were resolved by consensus, in consultation with another reviewer (D.M.). The database queries did not yield records that had to be excluded for language reasons, although 1 of the selection criteria was publication language (only articles published in English, German, French, Italian, Spanish, Portuguese, Russian, and Ukrainian were included).

### Data Extraction and Quality Assessment

Data were extracted using a standardized extraction form. This form sought information on publication details (eg, title), study design and population, participant characteristics, description of intervention, and outcomes.

Two of us (A.G., M.F.) independently assessed risk of bias using validated tools, and discrepancies were resolved by consensus and involved a third reviewer if necessary. The Physiotherapy Evidence Database (PEDro) Scale was used for the risk-of-bias assessment of RCTs. The PEDro Scale is based on 11 criteria, which are scored ranging from 0 to 10, with higher values indicating higher quality (less risk of bias).^6^ Risk of bias of nonrandomized controlled trials, cohort studies, and case-control studies was assessed using the Cochrane Risk-of-Bias in Non-randomized Studies of Intervention (ROBINS-I) tool.^7^ The ROBINS-I tool includes 7 domains (confounding, selection, classification of interventions, deviations from intended interventions, missing data, outcome measurement, and reporting) that are assessed as having low, moderate, high, or critical risk of bias.

### Outcome Measures and Data Synthesis

Primary outcomes were the number of postoperative complications, 30-day ED visits, and 30-day hospital readmissions. Secondary outcomes were patient satisfaction with digital health interventions and hospital length of stay (LOS), which was used as a proxy for the occurrence of adverse events.

### Statistical Analysis

For the primary outcomes, which were reported as contingency tables, risk ratios (RRs) comparing the telemedicine and conventional care groups were calculated. For LOS (a secondary outcome), standardized mean differences (SMDs) were calculated between the treatment and control groups. The SMDs were used instead of the raw differences due to variations in measurement scales across studies. The calculation and interpretation of SMD are described in the eMethods in Supplement 1. The meta-analytic RR estimates and their corresponding 95% CIs were obtained with a random-effects model and Mantel-Haenszel test. Der Simonian-Laird estimates were used as the default option of the RevMan software (Cochrane Collaboration) (eMethods in Supplement 1). The meta-analytic estimates were stratified by study design (RCTs vs non-RCTs) to assess the magnitude of potential biases due to nonrandomization of participants. In addition, subgroup analyses by type of telemedicine intervention were performed for primary outcomes. Heterogeneity of effect sizes was assessed using the *I*^2^ statistic. The number needed to treat (NNT) was estimated using the formulae described by McQuay et al.^8^

The level of significance for all tests was set to *P* = .05. Analyses were performed using RevMan v.5.4 (Cochrane Collaboration) and the R environment (R Project for Statistical Computing). The results concerning patient satisfaction (a secondary outcome) were summarized in narrative form because the measures in the original studies were not comparable and, therefore, not amenable to a quantitative analysis.

## Results

**Figure 1.**
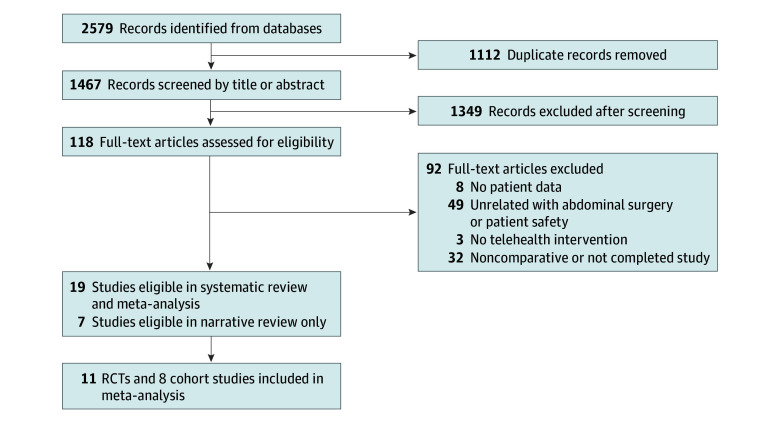
PRISMA Flow Diagram of Study Search and Selection RCT indicates randomized clinical trial.

The search and selection flowchart is presented in Figure 1. A total of 2579 records were identified in the initial search, and 1467 records remained after removal of duplicates. Of the 118 articles assessed for eligibility, 19 were eligible for the systematic review and meta-analysis and another 7 for the narrative review only. Finally, 11 RCTs^9,10,11,12,13,14,15,16,17,18,19,20^ and 8 cohort studies^21,22,23,24,25,26,27,28^ were included in this meta-analysis.

### Study Characteristics

The selected studies were published between 2013 and 2023 (Table). Fourteen (73.7%) of these studies were from North America (US and Canada),^10,12,14,15,16,17,18,21,23,24,25,26,27,28^ and the other 5 (26.3%) were from the UK, Australia, the Netherlands, Sweden, and Spain.^9,11,13,19,20,22^ The studies selected for the current meta-analysis included a total of 10 536 patients (3885 in the RCTs and 6651 in the nonrandomized cohort studies). In the RCTs, 1946 patients were in the telemedicine group and 1939 were in the conventional care group. The non-randomized studies included 3376 patients in the telemedicine cohort and 3275 in the conventional care group. Abdominal surgical procedures were represented in 7 studies (36.8%),^9,19,22,24,25,26,28^ 7 studies (36.8%) included patients who underwent colorectal procedures,^10,15,17,20,21,23,27^ 3 studies (15.8%) recruited patients who underwent gynecological procedures,^12,14,18^ and 2 studies (10.5%) included patients with different types of surgical procedures.^11,16^

**Table.** Studies Included in the Meta-Analysis

Source	Study design	Sample size (n = telemedicine group; conventional care group)	Type of intervention	Description of intervention	Type of surgery
Bednarski et al,^10^ 2019	RCT	30 (14; 16)	Mobile application for follow-up or recovery program	Monitoring of recovery via mobile application messaging with surgical team and videoconferencing, in addition to regular ERAS program	Colorectal
Cremades et al,^9^ 2020	RCT	200 (100; 100)	Telemedicine consultation	Follow-up via video call, with clinical and wound condition assessed and pathology discussed	Abdominal
Dahlberg et al,^11^ 2017 and Jaensson et al,^13^ 2017	RCT	997 (494; 503)	Mobile application for follow-up or recovery program	Smartphone application assessment of postoperative recovery using the symptoms questionnaire system, with daily reminder and contact with surgery team via the application	Different types
Halder et al,^12^ 2022	RCT	143 (70; 73)	Telemedicine consultation	Preoperative scripted telephone call, based on the enhanced-recovery program	Gynecological
Lee et al,^14^ 2021	RCT	54 (27; 27)	Telemedicine consultation	Virtual video follow-up visits at 3-4 d after discharge and 30 postoperative days	Gynecological
Mata et al,^15^ 2020	RCT	100 (50; 47)	Mobile application for follow-up or recovery program	Mobile application for postoperative education and self-assessment of recovery; the application included 3 sections: milestones checklist, daily clinical questionnaire, and education	Colorectal
McGilion et al,^16^ 2021	RCT	905 (451; 454)	Telemetry or telediagnostic	Remote monitoring technology measurement of biophysical variables and completion of daily recovery survey; participants interacted daily with a nurse virtually through the tablet	Different types
Pooni et al,^17^ 2022	RCT	253 (128; 125)	Mobile application for follow-up or recovery program	Postdischarge monitoring with the application, which included daily health check-up questions, image-uploading module for wound and stoma photos, and self-management postoperative recommendations	Colorectal
Thompson et al,^18^ 2019	RCT	103 (52; 51)	Telemedicine consultation	Scripted telephone follow-up to review specific assessment criteria, including evaluation of pain, bowel and bladder function, and return to daily activities	Gynecological
van der Meij et al,^19^ 2018	RCT	344 (173; 171)	Mobile application for follow-up or recovery program	Mobile application consisting of a personalized recovery plan, educational information, activity tracking, and recovery monitoring, with functions of active feedback and e-consultation	Abdominal
Young et al,^20^ 2013	RCT	756 (387; 369)	Telemedicine consultation	Five scheduled, structured telephone calls on days 3 and 10 and then at months 1, 3, and 6 after hospital discharge; included 22 standardized screening questions	Colorectal
Borsuk et al,^21^ 2019	Nonrandomized	281 (168; 113)	Mobile application for follow-up or recovery program	Mobile application for home prerehabilitation or rehabilitation and monitoring, including text messages with recommendations to recovery, patient-reported outcomes collection, and contacts with treatment team	Colorectal
Daliya et al,^22^ 2022	Nonrandomized	510 (210; 300)	Telemedicine consultation	Digital follow-up via videoconferencing or telephone follow-up	Abdominal
Eustache et al,^23^ 2023	Nonrandomized	350 (94; 256)	Mobile application for follow-up or recovery program	Mobile application with patient education material, daily questionnaires assessing postdischarge recovery, and patient-clinician chat function	Colorectal
Liu et al,^24^ 2021	Nonrandomized	2009 (1688; 321)	Telemedicine consultation	Follow-up telephone call after inguinal hernia surgery	Abdominal
Lovasik et al,^25^ 2020	Nonrandomized	528 (217; 311)	Telemedicine consultation	Follow-up telephone call, with additional call for high-risk patients	Abdominal
Nikolian et al,^26^ 2018	Nonrandomized	718 (233; 485)	Telemedicine consultation	Secure videoconferencing on cellular phone, tablet, or laptop, using the virtual medical platform	Abdominal
Stapler et al,^27^ 2022	Nonrandomized	1720 (668; 1052)	Mobile application for follow-up or recovery program	Extended recovery program included postoperative monitoring using a mobile application, with health status and wound care questionnaire and physical therapy and nutrition advice	Colorectal
Uppal et al,^28^ 2022	Nonrandomized	535 (98; 437)	Telemedicine consultation	Virtual, video, or audio contact for the first follow-up visit after hospital discharge	Abdominal

Abbreviations: ERAS, Enhanced Recovery After Surgery; RCT, randomized clinical trial.

The risk-of-bias assessment of the RCTs using the PEDro Scale revealed that the risk of bias was mainly due to the inability to blind participants, therapists, and assessors. The main source of the risk of bias in non-randomized studies assessed with the ROBINS-I tool was the selection of participants given that most cohort studies were retrospective and the selection process was not fully clarified. Another common source of potential bias was missing data due to the authors’ reported inability to completely follow up patients’ visits to other clinicians.^22,24,25,26,28^ The risk-of-bias assessment results are provided in eFigures 1 and 2 in Supplement 1.

### Primary Outcomes

**Figure 2.**
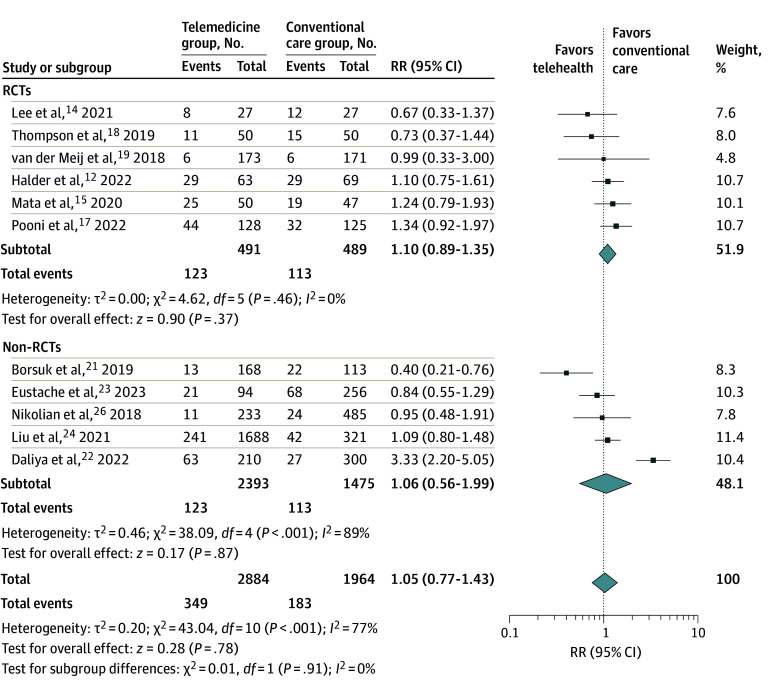
Complications of All Studies and Subgroups Error bars represent 95% CIs, square sizes represent the weight of the study, and diamonds represent the pooled risk ratio (RR) estimate (the width of the diamond indicates the 95% CI of the pooled RR). RCT indicates randomized clinical trial.

Eleven of 19 studies (57.9%) reported the occurrence of complications as an outcome.^12,14,15,17,18,19,21,22,23,24,26^ There was no significant difference in complication rate between the telemedicine group and the conventional care group (16.4% vs 15.1%). The meta-analytic RR estimate (Figure 2) did not suggest any differences between the 2 groups (RR, 1.05; 95% CI, 0.77-1.43). The heterogeneity among these studies for complication rate was large (*I*^2^ = 77%). Subgroup analysis comparing RCTs vs non-randomized studies did not suggest any differences in meta-analytic RR estimates (1.10 [95% CI, 0.89-1.35] vs 1.06 [95% CI, 0.56-1.99]).

**Figure 3.**
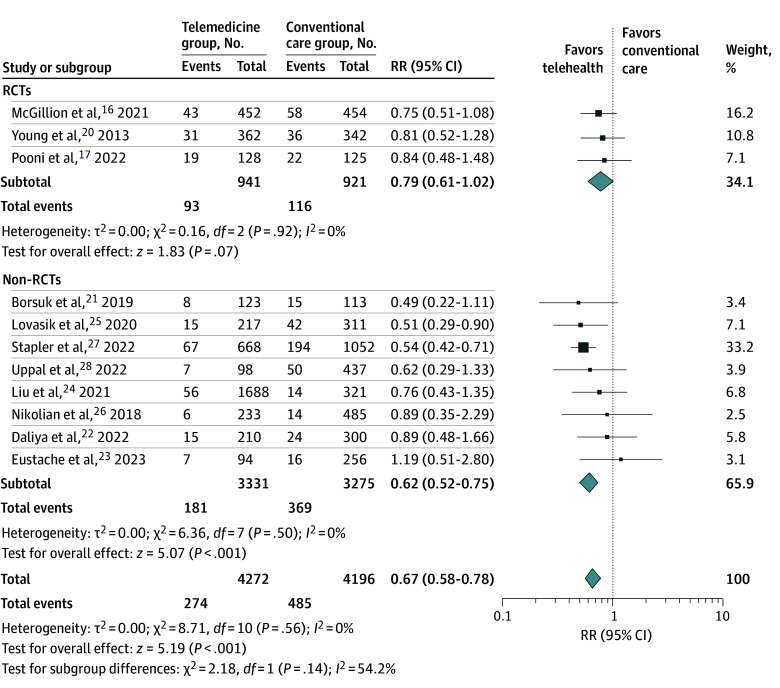
Readmissions of All Studies and Subgroups Error bars represent 95% CIs, square sizes represent the weight of the study, and diamonds represent the pooled risk ratio (RR) estimate (the width of the diamond indicates the 95% CI of the pooled RR). RCT indicates randomized clinical trial.

Rates of hospital readmissions were reported in 11 studies (Figure 3).^16,17,20,21,22,23,24,25,26,27,28^ A lower incidence of readmissions was observed in the telemedicine group (RR, 0.67; 95% CI, 0.58-0.78) with low heterogeneity (*I*^2^ = 0%). However, whereas differences in the RCT subgroup were not substantial (RR, 0.79; 95% CI, 0.61-1.02), the meta-analytic RR estimates obtained from nonrandomized studies revealed a significant reduction in readmission rates (RR, 0.62; 95% CI, 0.52-0.75). Nonetheless, in an additional moderation analysis, the differences between RCTs and non-randomized studies were not significant, suggesting a similar direction in associations.

**Figure 4.**
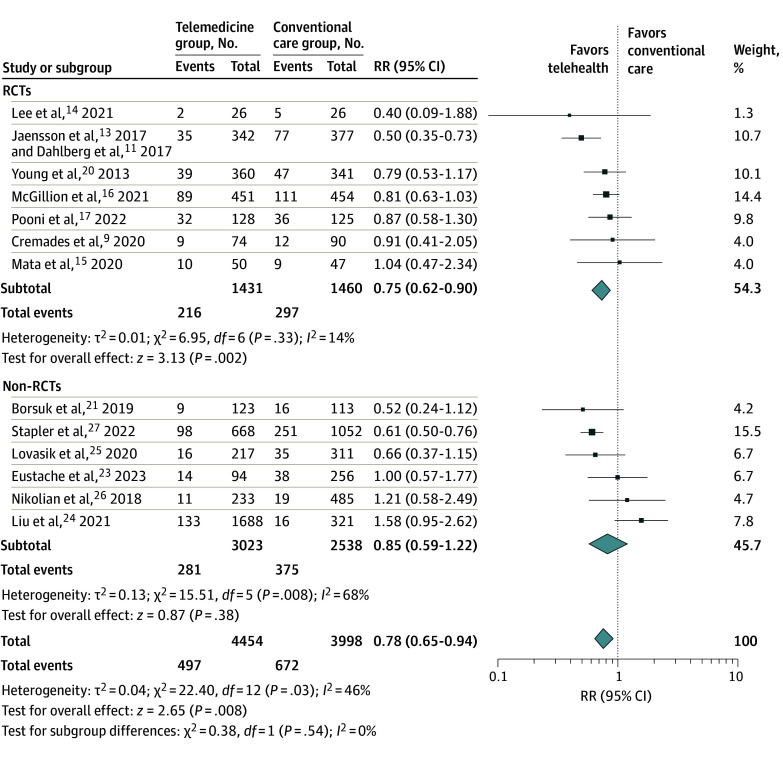
Emergency Department Visits of All Studies and Subgroups Error bars represent 95% CIs, square sizes represent the weight of the study, and diamonds represent the pooled risk ratio (RR) estimate (the width of the diamond indicates the 95% CI of the pooled RR). RCT indicates randomized clinical trial.

Seven RCTs and 6 nonrandomized cohort studies reported appropriate data on ED visits (Figure 4).^9,11,14,15,16,17,20,21,23,24,25,26,27^ The pooled estimate for all studies revealed a decrease in the number of ED visits in the telemedicine group (RR, 0.78; 95% CI, 0.65-0.94). This result showed moderate heterogeneity (*I*^2^ = 46%). Analysis of the RCTs also demonstrated a reduction in ED visits in favor of the telemedicine group (RR, 0.75; 95% CI, 0.62-0.90).

Subgroup analysis for RCTs with NNT calculations showed a small to medium effect size (NNT = 37 [95% CI, –640 to 17] for avoiding readmissions; NNT = 19 [95% CI, 12-40] for avoiding ED visits) in comparing telemedicine interventions with conventional care (eTable 1 in Supplement 1). Negative NNT values corresponded to the number needed to harm.

### Secondary Outcomes

Nine studies reported LOS as an outcome,^10,14,15,16,17,21,23,26,28^ of which 7 presented data appropriate for meta-analytic synthesis (eFigure 3 in Supplement 1). Five studies reported a decrease in LOS in the telemedicine group.^10,21,23,26,28^ Two of these studies focused primarily on reducing LOS with an enhanced postoperative recovery and monitoring program.^10,21^ The pooled estimate of the SMD showed a significant shortening of LOS in the telehealth group (SMD, −0.43 [95% CI, −0.65 to −0.22] days). The overall heterogeneity was high (*I*^2^ = 74%).

Fourteen studies presented different types of patient satisfaction measures.^9,10,12,14,15,17,18,19,20,23,26,29,30,31^ Patient satisfaction data are available in eTable 2 in Supplement 1. Overall, the studies reported high levels of patient compliance and satisfaction with telehealth technologies in perioperative management. Patient feedback was related to overall satisfaction with treatment, discharge, and follow-up after surgery as well as to usability and satisfaction with the telemedicine application. Most studies compared patient satisfaction in the telemedicine intervention and conventional care groups. Three of them showed significant advantages with telemedicine over conventional care,^12,17,31^ while the others found no difference between the groups. Based on qualitative feedback and patient comments, the most frequently cited advantages were ease of contact with the surgical team and cost and time savings from traveling to the site for follow-up.^14,23,26^ Furthermore, in some instances, patients were willing to recommend telehealth as an alternative to the traditional approach.^23,26,31^

### Types of Interventions

We found 3 types of interventions in the included studies: (1) telemedicine consultations via video call^9,14,22,25,26,28^ or scripted telephone call,^18,20,24,25^ which were conducted in 10 studies; (2) perioperative recovery program, patient education, follow-up, and health monitoring delivered via a mobile application, which was used in 8 studies^10,11,15,17,19,21,23,27^; and (3) remote monitoring of physiological variables using a telemedicine device, which was conducted in 1 study.^16^ Subgroup analysis by intervention type was performed to assess the change in primary outcomes associated with methodological diversity (eTable 3 in Supplement 1). The results suggest that telemedicine consultation and use of mobile applications may play a role in reduced rates of readmission; whereas for ED visits, only mobile applications seemed to be associated with lower rates.

## Discussion

This systematic review and meta-analysis included 19 studies that assessed the implications of telemedicine interventions for complication and readmission rates in patients who underwent abdominal surgery. There was no difference in complication rates in favor of telemedicine intervention compared with conventional care, and heterogeneity for this outcome was high. The pooled RR estimates showed a reduction in readmission rates and ED visits in the telemedicine group compared with conventional care group, with low to moderate heterogeneity.

Most telemedicine interventions consisted of consultations via video calls^9,14,22,25,26,28^; scripted telephone calls^18,20,24,25^; and mobile applications^11,15,17,19,21,23,27^ of varying functionality, including messaging, videoconferencing, perioperative education, monitoring of symptoms and recovery, and activity tracking. Thus, the most common feature of digital health interventions seemed to be the facilitation of communication between patients and health professionals with different levels of information, including clinical parameters (symptoms monitoring), feedback (face-to-face consultation and messaging), and knowledge transmission (education and training). Given the multifaceted character of the technologies implemented in the telemedicine interventions themselves and the lack of information on how each facet may be associated with patient safety outcomes, surgery types, and patient characteristics, it is not feasible to draw a conclusion on how digital health technologies play a role in the reduced burden of complications in abdominal surgery.

For instance, the reduction in the number of readmissions and ED visits may be associated with patients’ or clinicians’ assessment of the improved severity of symptoms either via the messaging system, video calls, postoperative education, or all of those components together. A similar difficulty can arise concerning telemedicine’s associated complication rates, since the expected reduction in the number of complications may be difficult to detect due to substantial heterogeneity in assessment methods and patient populations. Thus, Daliya et al^22^ found more complications in their telemedicine care group than the no follow-up group. However, on closer inspection, the increased complication rate was associated with more accurate and frequent recording of even minor events, which are not reported by patients in traditional postoperative follow-up. This finding seemed to point out that digital technology for reporting complications (eg, mobile applications and telemedicine consultations) is associated with increased probability of reporting less severe complications compared with nondigital procedures. Given the lack of a detailed qualification of the severity of complications in the included studies, the findings from the present study suggest that the complex interactions among the reporting behavior of patients, degree of seriousness and severity of complications, and functionality of digital technologies need further scrutiny.

The role of telehealth in the decreased incidence of complications may depend on the selected patient populations. For example, if the overall rate of complications is usually low after a minor 1-day procedure, such as cholecystectomy or hernioplasty, the outcome of telemedicine would also be difficult to observe.^32^ On the other hand, after major surgery, such as multivisceral and hepatopancreatobiliary surgical procedures, the rate of complications requiring hospitalization is usually higher and may offset the potential effectiveness of telehealth interventions. However, a retrospective study by Lovasik et al^25^ of a single hepatopancreatobiliary surgeon’s practice showed a 76% reduction in 30-day readmission rates after implementation of a program for perioperative patient education and structured telephone symptom assessment after discharge. Lovasik et al^25^ attributed this outcome to early detection and reduction in avoidable readmissions.

Additionally, the considerable heterogeneity in the design of telemedicine interventions (eg, services delivered by telephone or video call and by mobile applications with different functionalities) impedes a more detailed analysis of the most effective types of telemedicine services. Although some telehealth interventions may focus only on postoperative follow-up and detection of complications, others may also include patient education materials and individualized recovery programs. These factors can affect the patient’s experience of use and, consequently, their adherence to treatment and the effectiveness of the intervention.

The implications of telemedicine for LOS may be associated with clinicians discharging patients earlier based on the ability to monitor patients’ postdischarge health status using a web application, along with the potential for patients to use the application to report developing symptoms. However, given that some telehealth interventions started before admission and some after discharge, further research is needed.

### Strengths and Limitations

The strength of this systematic review and meta-analysis is that, to our knowledge, it was the first to provide an overview of the implications of digital medical interventions for patient safety in abdominal surgery. However, there are some limitations to this study that warrant some caution in the interpretation of results.

First, we included some nonrandomized studies whose results were more prone to confounding than those of the RCTs. However, for the primary outcomes, we found that the significance and direction of the effect sizes were consistent in both RCTs and nonrandomized studies, despite differences in the point estimates. Furthermore, retrospective design and large samples of observational studies have ecological validity and provide additional information on how different technologies and settings may affect the outcomes. In addition, some nonrandomized studies have an appropriate prospective design, sufficient power, and adjustments for several potential sources of bias.^21,23,27^ Second, it was not possible to identify the specific mechanisms of action underlying the observed differences between telemedicine and conventional care groups; however, this study provided some indications of specific mechanisms that may inform future research. For instance, the use of mobile applications can reduce the time delays between complication onset and treatment; increase the monitoring of the recovery process (particularly in the first days after hospital discharge); or provide a more informative remote assessment of the patient’s status via pictures, videoconferences, and other media. Therefore, findings from the present study provide some avenues for future research that focus on specific mechanisms, which may be more effective in reducing adverse events in abdominal surgery.

## Conclusions

In this systematic review and meta-analysis of 19 RCTs and nonrandomized studies, decreased rates of 30-day readmissions and ED visits were found in the telemedicine group vs the conventional care group after abdominal surgical procedures. There was no reduction in complication rates or LOS. However, the specific mechanisms of action for particular types of abdominal surgery are still largely unknown, warranting further research.

## Notes

Supplement 1. **eAppendix.** Search Strategy
eMethods.
**eFigure 1.** RCTs Risk of Bias Assessment (PEDro Scale) **eFigure 2.** Non-RCTs Risk of Bias Assessment (ROBINS-I Tool) **eFigure 3.** Length of Hospital Stay of All Studies and Subgroups **eTable 1.** Number Needed to Treat, Randomized Controlled Studies **eTable 2.** Patient Satisfaction for All Studies **eTable 3.** Subgroup Analysis by Type of Intervention
eReferences.

Supplement 2.
Data Sharing Statement
