# Supplementary material for: Digital Health Interventions and Patient Safety in Abdominal Surgery: A Systematic Review and Meta-Analysis
Source: JAMA Netw Open. 2024 Apr 26;7(4):e248555. doi: 10.1001/jamanetworkopen.2024.8555 (PMC11053376; doi:10.1001/jamanetworkopen.2024.8555)
Supplement: Supplement 1. — eAppendix. Search Strategy eMethods. eFigure 1. RCTs Risk of Bias Assessment (PEDro Scale) eFigure 2. Non-RCTs Risk of Bias Assessment (ROBINS-I Tool) eFigure 3. Length of Hospital Stay of All Studies and Subgroups eTable 1. Number Needed to Treat, Randomized Controlled Studies eTable 2. Patient Satisfaction for All Studies eTable 3. Subgroup Analysis by Type of Intervention eReferences. [file jamanetwopen-e248555-s001.pdf]

Supplemental Online Content

Grygorian A, Montano D, Shojaa M, Ferencak M, Schmitz N. Digital health interventions and patient safety in abdominal surgery:
a systematic review and meta-analysis. *JAMA Netw Open*. 2024;7(4):e248555.
doi:10.1001/jamanetworkopen.2024.8555

eAppendix. Search Strategy

eMethods.

eFigure 1. RCTs Risk of Bias Assessment (PEDro Scale)

eFigure 2. Non-RCTs Risk of Bias Assessment (ROBINS-I Tool)

eFigure 3. Length of Hospital Stay of All Studies and Subgroups

eTable 1. Number Needed to Treat, Randomized Controlled Studies

eTable 2. Patient Satisfaction for All Studies

eTable 3. Subgroup Analysis by Type of Intervention

eReferences.

This supplemental material has been provided by the authors to give readers additional information about their work.

© 2024 Grygorian A et al. *JAMA Network Open*

eAppendix. Search strategy

A systematic literature search was conducted through the MEDLINE (via PubMed and OVID), EBM/Cochrane, and Web of Science databases for all articles published up to February 02, 2023.

The search strategy will be utilized the population, intervention and outcome approach. The literature search will be constructed around search terms for “Telemedicine”, “Patient safety”, and “Surgery”.

A standard protocol for this search is being developed and controlled vocabulary (MeSH term for MEDLINE) will be used. We will use Key words and their synonymous to sensitize the search by applying the following concept:

The search strategy for PUBMED

search	Query
#1	Telemedicine[MeSH Terms]
#2	((Telemedicine[Title/Abstract]) OR (Telemedicine[Text Word] OR Telehealth[Text Word] OR "Mobile Health"[Text Word] OR mHealth[Text Word] OR eHealth[Text Word] OR "Digital health"[Text Word] OR Telecommunication*[Text Word] OR "Remote consultation*"[Text Word] OR "Remote monitoring"[Text Word] OR "Health information technology"[Text Word] OR videoconferencing[Text Word] OR "Video?consultation*"[Text Word]))
#3	(“App”[Text Word] OR software[Text Word] OR mobile phone[Text Word] OR smartphone[Text Word] OR cell phone[Text Word] OR "mobile?device" [Text Word])
#4	#1 OR #2 OR #3
#5	Patient Safety[MeSH Terms]
#6	Patient Outcome Assessment[MeSH Terms] OR Outcome Assessment[MeSH Terms]
#7	(“Patient* Safety” [Title/Abstract]) OR (“Patient* Safety”[Text Word] OR “Patient Outcome Assessment”[Text Word] OR Outcome*[Text Word] OR “follow?up”[Text Word] OR prevention[Text Word] OR perioperative[Text Word] OR postoperative[Text Word] OR “Surgical site infection”[Text Word] OR readmission[Text Word] OR Complication*[Text Word] OR disability[Text Word] OR “Adverse events”[Text Word] OR outpatient[Text Word] OR inpatient[Text Word] OR Consequen*[Text Word] OR “After discharge”[Text Word] OR “Post?discharge”[Text Word] OR control[Text Word] OR "Patient* reported" [Text Word])
#8	#5 OR #6 OR #7
#9	General surgery[MeSH Terms]
#10	(“Surgery” [Title/Abstract]) OR (“Abdominal surgery”[Text Word] OR Abdominal[Text Word] OR Gynecolog*[Text Word] OR Colorectal[Text Word] OR Hernia[Text Word] OR Bariatric[Text Word] OR “Weight?loss”[Text Word] OR Coloproctolog*[Text Word] OR “Abdominal wall”[Text Word] OR Laparoscop*[Text Word] OR “surgical procedure*”[Text Word] OR “surgical operation*”[Text Word] OR operative[Text Word])
#11	#9 OR #10
#12	#4 AND #8 AND #11

© 2024 Grygorian A et al. *JAMA Network Open*

eMethods.

For LOS (secondary outcome), standardized mean differences (SMD) were calculated between the treatment and control groups.

We used the standardized mean differences, instead of the raw differences, because the scale of measurement differed across studies, e.g., some reported hours, others number of nights. The SMD ensures comparability across studies.

To calculate SMD, the mean difference between the treatment and control groups in each study should be divided by that study's standard deviation[33].

The SMD can be easily interpreted in terms of any LOS scale as the product d*SD, where SD is the standard deviation of the LOS scale (e.g., hours, days, etc).

The meta-analytic RR estimates and the corresponding 95% confidence intervals were obtained with a random-effects model and Mantel– Haenszel test. Der Simonian-Laird estimates were used as the default option of the RevMan software.

There are several methods to estimate the random-effects models. Despite the existing criticism of the Der Simonian-Liard method, studies comparing different methods (Hunter-Schmidt, restricted maximum likelihood REML, Sidik-Jonkman, etc.) have shown that Der Simonian estimator is not per se inappropriate[34,35]

For our study we found concordant results for several estimators. Here we just report as an illustration the REML and the Sidik-Jonkman (SJ) estimators, both with the Knapp and Hartung method for the test statistic and confidence interval:

Complications:
Der Simonian (as reported in the manuscript): 1.05 [0.77-1.43]; REML: 1.04 [0.73-1.49]; SJ: 1.04 [0.73-1.49].

Readmissions:
Der Simonian (as reported in the manuscript): 0.67 [0.58-0.78]; REML: 0.69 [0.58-0.81]; SJ: 0.7 [0.59-0.82]

Since the numerical differences are very small and they do not affect the conclusions, we opted for leaving the defaults of the RevMan software.

© 2024 Grygorian A et al. *JAMA Network Open*

Study ID	*Eligibility*	*Random allocation*	*Concealed allocation*	*Baseline comparability*	*Blinding subjects*	*Blinding therapists*	*Blinding assessors*	*Completed follow-up*	*Intention to treat concept*	*Between-group comparisons*	*Point estimated variability*	***PEDro score***
Mata (2020)	yes	1	1	1	0	1	1	1	1	1	1	**9**
VanDerMeij (2018)	yes	1	1	1	1	0	1	1	1	1	1	**9**
Dahlberg/Jaensson (2017)	yes	1	1	1	0	0	1	1	1	1	1	**8**
McGilion (2021)	yes	1	1	1	0	0	1	1	1	1	1	**8**
Young (2018)	yes	1	1	1	1	0	0	1	1	1	1	**8**
Cremades (2020)	yes	1	1	1	0	0	0	1	1	1	1	**7**
Bednarsky (2019)	yes	1	1	1	0	0	0	1	1	1	1	**7**
Thompson (2019)	yes	1	1	1	0	0	0	1	1	1	1	**7**
Halder (2022)	yes	1	0	1	0	0	0	1	1	1	1	**6**
Lee (2021)	yes	1	0	1	0	0	0	1	1	1	1	**6**
Pooni (2022)	yes	1	0	1	0	0	0	1	1	1	1	**6**

eFigure 1. RCTs risk of bias assessment (PEDro scale)

© 2024 Grygorian A et al. *JAMA Network Open*

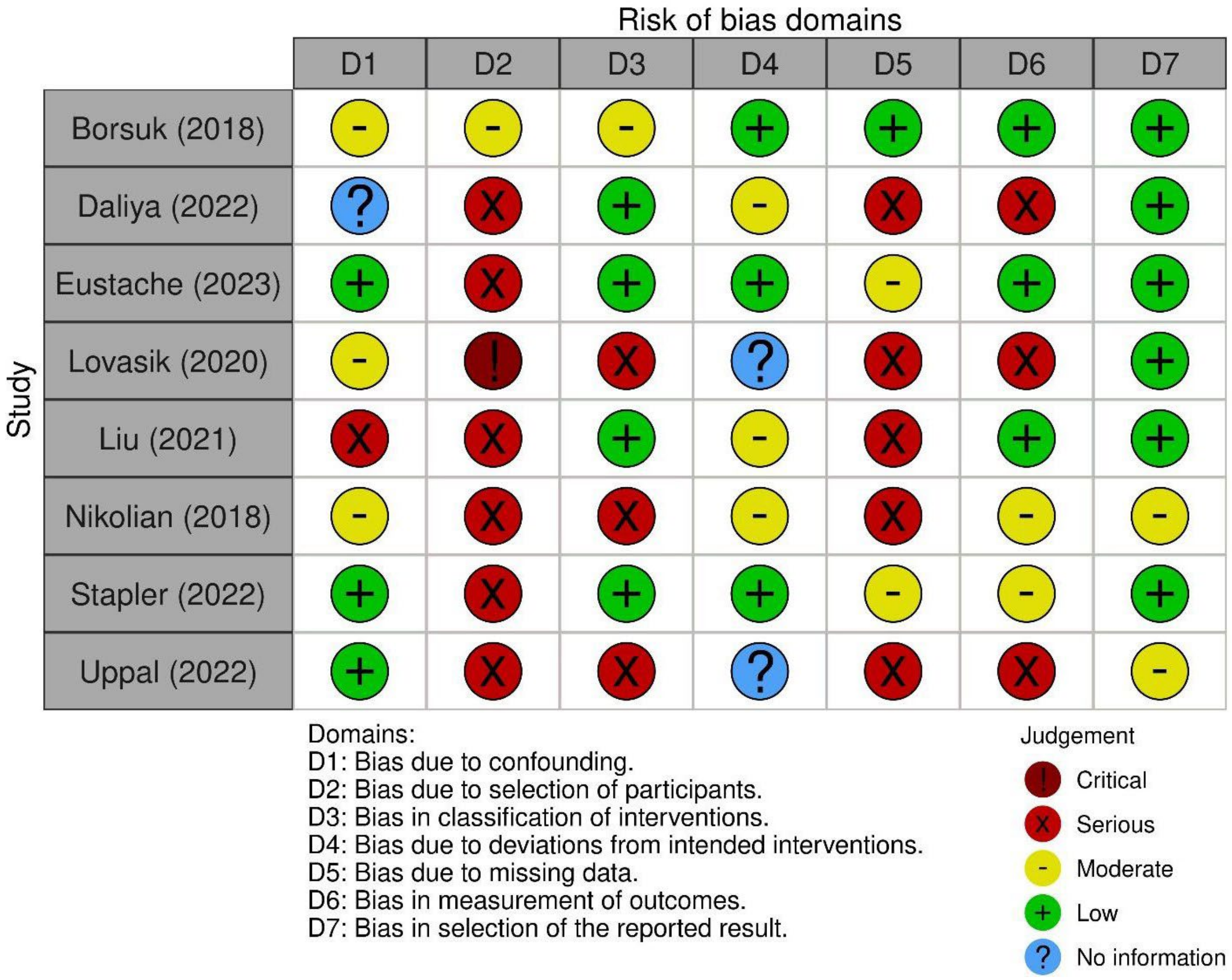

eFigure 2. Non-RCTs risk of bias assessment (ROBINS-I tool).

© 2024 Grygorian A et al. *JAMA Network Open*

Study or Subgroup	Telehealth Mean	Telehealth SD	Telehealth Total	Control Mean	Control SD	Control Total	Weight	Std. Mean Difference IV, Random, 95% CI
**5.1.1 RCTs**								
Mata (2020)	4.6	2.89	50	6.4	13.2	47	12.7%	-0.19 [-0.59, 0.21]
Gerceker (2016)	1.8	1.1	24	2.1	1.2	30	9.4%	-0.26 [-0.79, 0.28]
Bednarski (2019)	1.17	0.44	14	2.24	0.74	16	5.0%	-1.68 [-2.53, -0.83]
**Subtotal (95% CI)**			**88**			**93**	**27.1%**	**-0.62 [-1.36, 0.12]**
Heterogeneity: Tau² = 0.33; Chi² = 10.07, df = 2 (P = 0.007); I² = 80%								
Test for overall effect: Z = 1.64 (P = 0.10)								
**5.1.2 Non-RCTs**								
Uppal (2022)	3.22	2.02	98	4.25	2.89	437	18.1%	-0.37 [-0.59, -0.15]
Nikolian (2018)	1.21	0.77	233	1.41	0.87	485	19.9%	-0.24 [-0.39, -0.08]
Eustache (2023)	3.2	4.94	94	4.6	4.48	256	17.6%	-0.30 [-0.54, -0.07]
Borsuk (2018)	2.6	2.8	168	4.7	2.6	113	17.3%	-0.77 [-1.02, -0.52]
**Subtotal (95% CI)**			**593**			**1291**	**72.9%**	**-0.41 [-0.63, -0.19]**
Heterogeneity: Tau² = 0.04; Chi² = 13.08, df = 3 (P = 0.004); I² = 77%								
Test for overall effect: Z = 3.64 (P = 0.0003)								
**Total (95% CI)**			**681**			**1384**	**100.0%**	**-0.43 [-0.65, -0.22]**
Heterogeneity: Tau² = 0.06; Chi² = 23.18, df = 6 (P = 0.0007); I² = 74%								
Test for overall effect: Z = 3.92 (P < 0.0001)								
Test for subgroup differences: Chi² = 0.28, df = 1 (P = 0.60), I² = 0%								

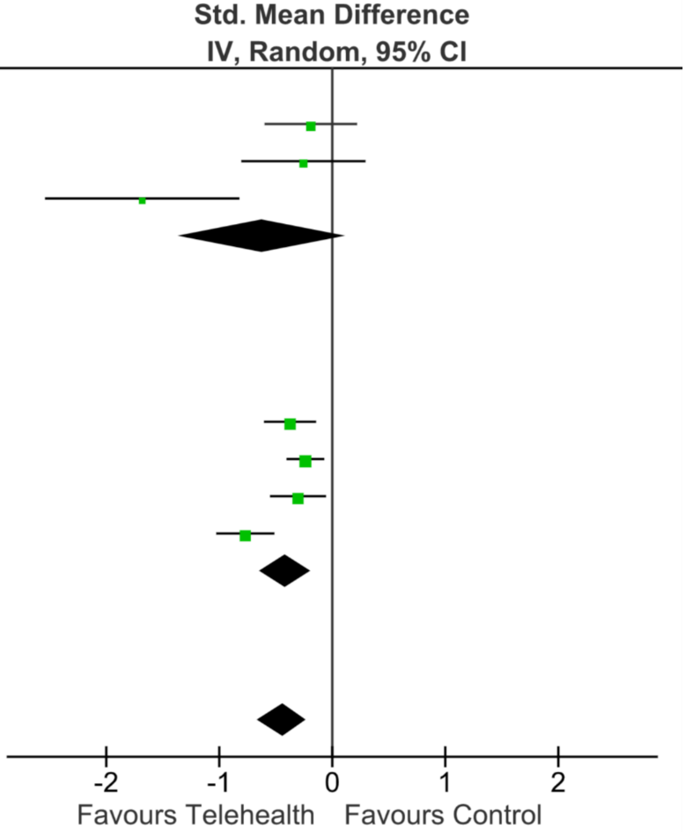

eFigure 3. Length of hospital stay of all studies and subgroups

© 2024 Grygorian A et al. *JAMA Network Open*

eTable 1. Number needed to treat, randomized controlled studies.

Readmissions									
	**Readmissions**	**No readmissions**	**Margins**	**Absolute risk**	**RR[a] control vs. telemedicine**	**Inverse RR**	**ARR[b] % Telemedicine-control**	**NNT[c]**	**Confidence interval NNT**
Control	116	805	921	0,13	1,27	0,78	2,7	37	[17; -640]
Telemedicine	93	848	941	0,10					
ED visits									
	**ED visits**	**No ED visits**	**Margins**	**Absolute risk**	**RR control vs. telemedicine**	**Inverse RR**	**ARR % Telemedicine – control**	**NNT**	**Confidence interval NNT**
Control	297	1163	1460	0,20	1,35	0,74	5,2	19	[12; 40]
Telemedicine	216	1215	1431	0,15					

[a]RR – relative risk, [b]ARR – absolute risk reduction [c]NNT - number needed to treat

© 2024 Grygorian A et al. *JAMA Network Open*

eTable 2. Patient satisfaction

Study ID	*Type of measure*	*Telehealth*	*Control*	*P-value*
Bednarski (2019)	20-question survey a multiple aspect of the perioperative care pathway. Overall satisfaction - number of patients answer "very satisfied", n (%) _	12 (92.3)	12 (85.7)	1
Cremades (2020)	UK NHS outpatient's questionnaire combined with the TUQ (Telehealth usability questionnaire). All the patients were asked to provide a global satisfaction score on a scale from 1 to 5. Median (range),	5 (1-5)	5 (2-5)	0.099
Eustache (2023)	Qualitative survey of app usability and satisfaction with 1 to 10 scale. How likely patients were to recommend the app to another patient undergoing surgery, patients scored the app, mean (SD)	9.4 (1.5)	na	na
Fink (2022)	Satisfaction level measured with 5-point Likert-scale, survey include 6 questions (satisfaction with surgery, with follow-up method, prefer another method, timing of consultation, recommendations to friends, overall satisfaction. Overall, % of satisfied	60%	49%	0.318
Goedeke (2018)	Satisfaction measured with a six-step scale "I was very satisfied with the consultation": 1 = strongly disagree, 6 = strongly agree, mean (95% CI)	5.40 (5.28-5.52)	5.10 (4.92-5.28)	0.029
Halder (2022)	Preoperative Preparedness Questionnaire (PPQ) question number 11 (Q11), "Overall, I feel prepared for my upcoming surgery." n (%) of patients feel prepared for surgery.	52 (83)	41 (59)	< 0.01
Lee (2021)	Patient Satisfaction Questionnaire-18, Mean (SD)	4.5 (0.4)	4.4 (0.4)	0.5
Ma (2018)	Satisfaction level was measured on a non-validated scale from 0 to 10 (0: completely unsatisfied, 5: neutral, 10: completely satisfied), mean (SD),	9.31 (1.31)	8.85 (1.36)	0.002
Mata (2020)	Questionnaire consists of 4 items from the Consumer Assessment of Healthcare Providers and Systems Surgical Care Survey (S-CAHPS), 1 to 5 scale, median (range)	4 (4-5)	4 (3.5-5)	0.7
Nikolian (2018)	eClinic Patient Experience Survey Results (Listed as Percent ''Satisfied'' and ''Very Satisfied'' on a 5-point Scale), n=34, overall satisfaction	83%	na	na
Pooni (2022)	Satisfaction with the discharge process, 10-points scale median (IQR)	9 (8-10)	8 (7-9)	< 0.001
Thompson (2019)	S-CHAPS questionnaire, rating of the surgeon: percentage of respondents giving a "top box" answer to the S-CAHPS questionnaires, % (95 CI) with a non-inferiority limit of 36.1	48.9 (38.4 - 59.4)	51.1 (4.06 - 61.6)	0.006
Vandermeij (2018)	Satisfaction of overall received care, questionnaire on a scale 1 to 10, Mean (SD)	7.5 (1.7)	7.1 (2.3)	0.169
Young (2013)	Quantitative survey. Number of patients satisfied by assistance of research nurse, n (%)	259 (79.4)	na	na

© 2024 Grygorian A et al. *JAMA Network Open*

eTable 3. Subgroup analysis by type of intervention

	Weight	*RR (95% CI) Telemedicine vs control*	*Heterogeneity*
1. Readmissions			
1.1 Telemedicine consultation	53.2%	0.74 [0.60, 0.90]	Tau² = 0.00; Chi² = 2.52, df = 6 (P = 0.87); I² = 0%
1.2 Telemedicine App	46.8%	0.66 [0.47, 0.93]	Tau² = 0.04; Chi² = 4.65, df = 3 (P = 0.20); I² = 36%
Total	100.0 %	0.67 [0.58, 0.78]	Tau² = 0.00; Chi² = 8.71, df = 10 (P = 0.56); I² = 0%
			Subgroup differences: Chi² = 0.28, df = 1 (P = 0.60), I² = 0%
2. ED Visits			
2.1 Telemedicine consultation	59.7%	0.81 [0.62, 1.06]	Tau² = 0.07; Chi² = 15.65, df = 7 (P = 0.03); I² = 55%
2.2 Telemedicine App	40.3%	0.74 [0.58, 0.94]	Tau² = 0.02; Chi² = 5.60, df = 4 (P = 0.23); I² = 29%
Total	100.0 %	0.78 [0.65, 0.94]	Tau² = 0.04; Chi² = 22.40, df = 12 (P = 0.03); I² = 46%
			Subgroup differences: Chi² = 0.28, df = 1 (P = 0.60), I² = 0%

© 2024 Grygorian A et al. *JAMA Network Open*

eReferences

1. Ghomrawi HMK, Holl JL, Abdullah F. Telemedicine in Surgery—Beyond a Pandemic Adaptation. *JAMA Surgery*. 2021;156(10):901-902. doi:10.1001/jamasurg.2021.2052

2. Asiri A, AlBishi S, AlMadani W, ElMetwally A, Househ M. The Use of Telemedicine in Surgical Care: a Systematic Review. *Acta Inform Med*. Oct 2018;26(3):201-206. doi:10.5455/aim.2018.26.201-206

3. McLean KA, Knight SR, Diehl TM, et al. Readiness for implementation of novel digital health interventions for postoperative monitoring: a systematic review and clinical innovation network analysis. *Lancet Digit Health*. May 2023;5(5):e295-e315. doi:10.1016/S2589-7500(23)00026-2

4. Williams AM, Bhatti UF, Alam HB, Nikolian VC. The role of telemedicine in postoperative care. *Mhealth*. 2018;4:11. doi:10.21037/mhealth.2018.04.03

5. Classification of digital health interventions. Geneva: World Health Organization; 2018(WHO/RHR/18.06). Licence: CC BY-NC-SA 3.0 IGO.

6. de Morton NA. The PEDro scale is a valid measure of the methodological quality of clinical trials: a demographic study. *Aust J Physiother*. 2009;55(2):129-33. doi:10.1016/s0004-9514(09)70043-1

7. Sterne JA, Hernán MA, Reeves BC, et al. ROBINS-I: a tool for assessing risk of bias in non-randomised studies of interventions. *BMJ*. 2016;355:i4919. doi:10.1136/bmj.i4919

8. McQuay HJ, Moore RA. Using numerical results from systematic reviews in clinical practice. *Ann Intern Med*. May 1 1997;126(9):712-20. doi:10.7326/0003-4819-126-9-199705010-00007

9. Cremades M, Ferret G, Pares D, et al. Telemedicine to follow patients in a general surgery department. A randomized controlled trial. *Am J Surg*. Jun 2020;219(6):882-887. doi:10.1016/j.amjsurg.2020.03.023

10. Bednarski BK, Nickerson TP, You YN, et al. Randomized clinical trial of accelerated enhanced recovery after minimally invasive colorectal cancer surgery (RecoverMI trial). *Br J Surg*.

© 2024 Grygorian A et al. *JAMA Network Open*

Sep 2019;106(10):1311-1318. doi:10.1002/bjs.11223

11. Dahlberg K, Philipsson A, Hagberg L, Jaensson M, Halleberg-Nyman M, Nilsson U. Cost-effectiveness of a systematic e-assessed follow-up of postoperative recovery after day surgery: a multicentre randomized trial. *Br J Anaesth*. Nov 1 2017;119(5):1039-1046. doi:10.1093/bja/aex332

12. Halder GE, White AB, Brown HW, et al. A telehealth intervention to increase patient preparedness for surgery: a randomized trial. *Int Urogynecol J*. Jan 2022;33(1):85-93. doi:10.1007/s00192-021-04831-w

13. Jaensson M, Dahlberg K, Eriksson M, Nilsson U. Evaluation of postoperative recovery in day surgery patients using a mobile phone application: a multicentre randomized trial. *Br J Anaesth*. Nov 1 2017;119(5):1030-1038. doi:10.1093/bja/aex331

14. Lee DD, Arya LA, Andy UU, Harvie HS. Video Virtual Clinical Encounters Versus Office Visits for Postoperative Care After Pelvic Organ Prolapse Surgery: A Randomized Clinical Trial. *Female Pelvic Med Reconstr Surg*. Jul 1 2021;27(7):432-438. doi:10.1097/SPV.0000000000000909

15. Mata J, Pecorelli N, Kaneva P, et al. A mobile device application (app) to improve adherence to an enhanced recovery program for colorectal surgery: a randomized controlled trial. *Surg Endosc*. Feb 2020;34(2):742-751. doi:10.1007/s00464-019-06823-w

16. McGillion MH, Parlow J, Borges FK, et al. Post-discharge after surgery Virtual Care with Remote Automated Monitoring-1 (PVC-RAM-1) technology versus standard care: randomised controlled trial. *BMJ*. Sep 30 2021;374:n2209. doi:10.1136/bmj.n2209

17. Pooni A, Brar MS, Anpalagan T, et al. Home to Stay: A Randomized Controlled Trial Evaluating the Effect of a Post-discharge Mobile App to Reduce 30- Day Re-admission Following Elective Colorectal Surgery. *Ann Surg*. Jul 11 2022;doi:10.1097/SLA.0000000000005527

18. Thompson JC, Cichowski SB, Rogers RG, et al. Outpatient visits versus telephone interviews for postoperative care: a randomized controlled trial. *Int Urogynecol J*. Oct 2019;30(10):1639-1646. doi:10.1007/s00192-019-03895-z

19. van der Meij E, Anema JR, Leclercq WKG, et al. Personalised perioperative care by e-

© 2024 Grygorian A et al. *JAMA Network Open*

health after intermediate-grade abdominal surgery: a multicentre, single-blind, randomised, placebo-controlled trial. *Lancet*. Jul 7 2018;392(10141):51-59. doi:10.1016/S0140-6736(18)31113-9

20. Young JM, Butow PN, Walsh J, et al. Multicenter randomized trial of centralized nurse-led telephone-based care coordination to improve outcomes after surgical resection for colorectal cancer: the CONNECT intervention. *J Clin Oncol*. Oct 1 2013;31(28):3585-91. doi:10.1200/JCO.2012.48.1036

21. Borsuk DJ, Al-Khamis A, Geiser AJ, et al. S128: Active post discharge surveillance program as a part of Enhanced Recovery After Surgery protocol decreases emergency department visits and readmissions in colorectal patients. *Surg Endosc*. Nov 2019;33(11):3816-3827. doi:10.1007/s00464-019-06725-x

22. Daliya P, Carvell J, Rozentals J, Lobo DN, Parsons SL. Digital Follow-Up After Elective Laparoscopic Cholecystectomy: A Feasibility Study. *World J Surg*. Nov 2022;46(11):2648-2658. doi:10.1007/s00268-022-06684-w

23. Eustache JH, Latimer EA, Liberman AS, et al. A Mobile Phone App Improves Patient-Physician Communication and Reduces Emergency Department Visits After Colorectal Surgery. *Dis Colon Rectum*. Jan 1 2023;66(1):130-137. doi:10.1097/DCR.0000000000002187

24. Liu N, Greenberg JA, Xu Y, Shada AL, Funk LM, Lidor AO. Phone follow-up after inguinal hernia repair. *Surg Endosc*. Sep 2021;35(9):5159-5166. doi:10.1007/s00464-020-08005-5

25. Lovasik BP, Blair CM, Little LA, Sellers M, Sweeney JF, Sarmiento JM. Reduction in Post-Discharge Return to Acute Care in Hepatopancreatobiliary Surgery: Results of a Quality Improvement Initiative. *J Am Coll Surg*. Aug 2020;231(2):231-238. doi:10.1016/j.jamcollsurg.2020.03.034

26. Nikolian VC, Williams AM, Jacobs BN, et al. Pilot Study to Evaluate the Safety, Feasibility, and Financial Implications of a Postoperative Telemedicine Program. *Ann Surg*. Oct 2018;268(4):700-707. doi:10.1097/SLA.0000000000002931

© 2024 Grygorian A et al. *JAMA Network Open*

27. Stapler SJ, Brockhaus KK, Battaglia MA, Mahoney ST, McClure AM, Cleary RK. A Single-Institution Analysis of Targeted Colorectal Surgery Enhanced Recovery Pathway Strategies That Decrease Readmissions. *Dis Colon Rectum*. Jul 1 2022;65(7):e728-e740. doi:10.1097/DCR.0000000000002129

28. Uppal A, Kothari AN, Scally CP, et al. Adoption of Telemedicine for Postoperative Follow-Up After Inpatient Cancer-Related Surgery. *JCO Oncol Pract*. Jul 2022;18(7):e1091-e1099. doi:10.1200/OP.21.00819

29. Fink T, Chen Q, Chong L, Hii MW, Knowles B. Telemedicine versus face-to-face follow up in general surgery: a randomized controlled trial. *ANZ J Surg*. Oct 2022;92(10):2544-2550. doi:10.1111/ans.18028

30. Goedeke J, Ertl A, Zoller D, Rohleder S, Muensterer OJ. Telemedicine for pediatric surgical outpatient follow-up: A prospective, randomized single- center trial. *J Pediatr Surg*. Jan 2019;54(1):200-207. doi:10.1016/j.jpedsurg.2018.10.014

31. Ma Y JG, Tay YK, Hunter T, Holden D, Rodgers-Wilson S, Cashin P, Tan PY, Croagh D. Post-operative telephone review is safe and effective: prospective study - Monash outpatient review by phone trial. *ANZ J Surg* 2018 88(5):434-439. doi:doi: 10.1111/ans.14280

32. Hwa K, Wren SM. Telehealth follow-up in lieu of postoperative clinic visit for ambulatory surgery: results of a pilot program. *JAMA Surg*. Sep 2013;148(9):823-7. doi:10.1001/jamasurg.2013.2672

33. Borenstein, M. et al. Effect Sizes Based on Means. Introduction to Meta-Analysis. 2009:21-32. doi:10.1002/9780470743386.ch4

34. Montano D, Schleu JE, Hüffmeier J. A Meta-Analysis of the Relative Contribution of Leadership Styles to Followers' Mental Health. Journal of Leadership & Organizational Studies. 2023;30(1):90-107. doi:10.1177/15480518221114854

35. Thorlund K, Wetterslev J, Awad T, Thabane L, Gluud C. Comparison of statistical inferences from the DerSimonian–Laird and alternative random-effects model meta-analyses – an empirical assessment of 920 Cochrane primary outcome meta-analyses. Research Synthesis Methods. 2011;2(4):238-253. doi:10.1002/jrsm.53
