# Supplementary material for: Digital Health Interventions and Patient Safety in Abdominal Surgery: A Systematic Review and Meta-Analysis
Source: JAMA Netw Open. 2024 Apr 26;7(4):e248555. doi: 10.1001/jamanetworkopen.2024.8555 (PMC11053376; doi:10.1001/jamanetworkopen.2024.8555)
Supplement: Supplement 2. — Data Sharing Statement [file jamanetwopen-e248555-s002.pdf]

Data Sharing Statement

Grygorian. Digital Health Interventions and Patient Safety in Abdominal Surgery. *JAMA Netw Open*. Published April 26, 2024. doi:10.1001/jamanetworkopen.2024.8555

Data
Data available: Yes
Data types: Data dictionary
How to access data: Request from author Norbert.schmitz@med.Uni-Tübingen.de
When available: With publication

Supporting Documents
Document types: None

Additional Information
Who can access the data: Researchers
Types of analyses: Review
Mechanisms of data availability: Available upon request
